# An instrument based on protection motivation theory to predict Chinese adolescents’ intention to engage in protective behaviors against schistosomiasis

**DOI:** 10.1186/s41256-016-0015-6

**Published:** 2016-10-05

**Authors:** Han Xiao, Minjin Peng, Hong Yan, Mengting Gao, Jingjing Li, Bin Yu, Hanbo Wu, Shiyue Li

**Affiliations:** 1 School of Health Science, Wuhan University, Wuhan, Hubei China; 2 Xuanwu Hospital, Capital Medical University, Beijing, China; 3 Department of Infection Control, Taihe Hospital, Hubei University of Medicine, Shiyan, Hubei China; 4 Global Health Institute, Wuhan University, Wuhan, Hubei China; 5 Department of Behavioral Sciences and Health Education, Rollins School of Public Health, Emory University, Atlanta, Georgia, USA; 6 Department of Epidemiology, University of Florida, Gainesville, FL USA; 7 School of Demography, College of Arts and Social Science, The Australian National University, Canberra, ACT Australia

**Keywords:** Schistosomiasis, Protection Motivation Theory, Instrument, Adolescents

## Abstract

**Background:**

Further advancement in schistosomiasis prevention requires new tools to assess protective motivation, and promote innovative intervention program. This study aimed to develop and evaluate an instrument developed based on the Protection Motivation Theory (PMT) to predict protective behavior intention against schistosomiasis among adolescents in China.

**Methods:**

We developed the Schistosomiasis PMT Scale based on two appraisal pathways of protective motivation- threat appraisal pathway and coping appraisal pathway. Data from a large sample of middle school students (*n* = 2238, 51 % male, mean age 13.13 ± 1.10) recruited in Hubei, China was used to evaluated the validity and reliability of the scale.

**Results:**

The final scale contains 18 items with seven sub-constructs. Cronbach’s Alpha coefficients for the entire instrument was 0.76, and for the seven sub-constructs of severity, vulnerability, intrinsic reward, extrinsic reward, response efficacy, self-efficacy and response cost was 0.56, 0.82, 0.75, 0.80, 0.90, 0.72 and 0.70, respectively. The construct validity analysis revealed that the one level 7 sub-constructs model fitted data well (GFI = 0.98, CFI = 0.98, RMSEA = 0.03, Chi-sq/df = 3.90, *p* < 0.001). Predictive validity showed that both the PMT instrument score and the 7 sub-construct scores were significantly correlated with the intention engaged in protective behavior against schistosomiasis (*p* < 0.05).

**Conclusions:**

This study provides a reliable and valid tool to measure protective motivation in schistosomiasis prevention control. Further studies are needed to develop more effective intervention programs for schistosomiasis prevention.

## Background

### Schistosomiasis and health

Schistosomiasis, an acute and chronic parasitic disease in tropical and subtropical region, has been reported in 78 countries and at least 249 million people require preventive treatment all over the world in 2012 [1, 2]. It’s estimated that there are approximately 200,000 people infected with schistosomiasis per year, and the health cost of schistosomiasis is equivalent to HIV/AIDS, much higher than malaria and tuberculosis [3, 4]. Despite the great effort of Chinese government to control and prevent schistosomiasis, it still covers up to 5 % of the residents living in the epidemic area [5]. By the end of 2012, the number of schistosomiasis patients was 240,957 and 368,741.67 ha of the land was infested with *Oncomelania hupensis* snails, the intermediate host of schistosomiasis in China [6].

Schistosomiasis is an infectious disease, which is behavior-related. The infections are most likely to happen when humans come into contact with the schistosomiasis at miracidium stage in snail-conditioned water. [7, 8]. Thus, protective behaviors (avoiding contact with snail-conditioned water or engaging in protective measures when contact is unavoidable or required) are critical for controlling the schistosomiasis transmission. Adolescents are in a stage of imbalance between cognition and physical development, and numerous studies have reported higher prevalence of risk behaviors among adolescents. More risk-taking behavior and less protective behavior is expected among adolescents who live in the places where schistosomiasis is epidemic. Therefore, younger adolescents are at higher risk of schistosomiasis infections than other age groups across the world, as well as in China [2]. In addition, nearly half of the acute schistosomiasis infections are adolescents in China [9]. The rate of Chinese adolescents who do not correctly implement protective measures range from 32 to 56.8 %, suggesting the urgency to enhance Chinese adolescents’ behavioral prevention against schistosomiasis [10, 11].

### Knowledge-Attitude-Practice (KAP) Theory

The Knowledge-Attitude-Practice (KAP) Theory is commonly used as the guidance to schistosomiasis control and prevention in China. It emphasizes the knowledge of, correct attitudes towards schistosomiasis, as well as practical skills to reduce the likelihood of risk behaviors relating to schistosomiasis [12]. A three years health education program in Anhui, one of the heavy epidemic area in China, indicates that the awareness rate of schistosomasis related knowledge, attitudes towards this disease prevention and the compliance with examination and treatment of schistosomiasis among the workers were increased from 11.2, 9.1, 38.8 % and 31.7 % to 93.8, 91.9, 93.2 and 84.3 % through the intervention, respectively [13]. However, another intervention program in the same province showed that the rate of contacting with contaminated water did not decrease significantly while the awareness rate of schistosomasis and protective behavior related knowledge increased from 60 to 80 % [14]. These previous studies revealed that people’s knowledge of or correct attitudes toward schistosomiasis were improved after the intervention, but the proportion of people who engage in protective behaviors did not show consistent change [15–21]. The phenomenon of *“knowledge-practice”* separation is widely discussed in public health practice and research [12, 15, 16, 22, 23], and the challenge leads researchers to seek for alternative theories which are more effective to reduce adolescents’ risk behaviors.

### Protection Motivation Theory (PMT)

**Fig. 1:**
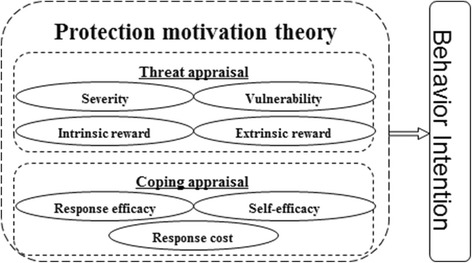
Frameworks of the Protection Motivation Theory and its seven sub-constructs

Generally, the intention of human beings to engage in any behavior is the most vital determinant of the behavior [24]. A social cognitive model named Protection Motivation Theory (PMT) has been developed and widely used in recent years to predict people’s intention to engage in protective behaviors [25–27]. In PMT theory, a man’s behavior depends on two correlated pathways, threat appraisal and coping appraisal: the threat appraisal pathway is an evaluation of a person’s perception of the threat of certain behaviors or diseases; the coping appraisal pathway is an evaluation of a person’s ability to cope with the threat. The threat appraisal pathway consists of two attributes, with two sub-constructs in each attribute. One attribute is the perceived threat with two sub-constructs (severity and vulnerability) and another attribute is the perceived rewards consisting of two sub-constructs (intrinsic rewards and extrinsic rewards). Coping appraisal pathway consists of two attributes. The first attribute assesses perceived efficacy, including two sub-constructs (i.e. response efficacy and self-efficacy). Another assesses perceived costs with one sub-constructs (i.e. response costs) (Fig. 1).

PMT has been widely adopted as a framework for the prediction of and intervention in a range of health-related behaviors [27]. An exploratory study for the correlation between Protection Motivation Theory measures and the intention to use drugs indicated that two component of the Theory (intrinsic and extrinsic rewards) explain nearly 50 % of the variation, with odds ratio of 2.90 and 8.04 (*p* < 0.05) [28]. As for smoking behavior, a series of studies revealed that perceived rewards of smoking, especially intrinsic rewards, were consistently positively related to smoking intentions and behavior, and self-efficacy to avoid smoking was negatively related to smoking [25, 26, 29–31]. Recently, Protection motivation was successfully applied for the prediction of preventing a group of infectious and chronic non-communicable diseases, such as HIV/AIDS [32, 33], Chlamydia [34], Respiratory Infectious Disease [35], Coronary Artery Disease [36], Cervical Pap Test Practice [37, 38] and other cancer prevention behaivors [39–43]. What’s more, tropical infectious diseases such as malaria, PMT has also been proved to be a powerful tool in analyzing malaria preventive behaviors [44]. However, as a serious tropical infectious diseases in China, few studies have applied the PMT in the schistosomiasis research. The application of PMT on schistosomiasis prevention was promising.

### Purpose of this study

Given the *“knowledge-practice”* separation in KAP theory and the strength of PMT theory in predicting protective behaviors, PMT theory could be applied to schistosomiasis prevention and control. However, to our knowledge, PMT haven’t been used in schistosomiasis control and prevention, and no such a measurement tool exists to assess the protective motivation about schistosomiasis. As the first step of applying PMT [26, 38, 41], the main purpose of this study is to develop and evaluate a PMT based schistosomiasis scale, and to investigate the association between components of PMT and protective behavior intention.

## Methods

### Study design and ethics statement

This research was conducted between September and October 2013 in Hubei province, China. The protocol was approved by Ethics Review Committee of Medical School, Wuhan University, China, and only participants who signed the informed consent were included in this study.

### Participants and sampling

Data were collected between September and October 2013. Participants were students attending middle schools from grade 7 to grade 9. Schools were selected from a highly endemic rural area in Hubei Province, China. Hubei, located in central China, is one of the provinces with the highest incidence rates [6].

Participants were selected using the stratified cluster sampling method. First, two counties were randomly selected from the epidemic-control area of Hubei province, and with the same sampling method, another two counties were selected from spread-control area. Epidemic-control area refers to the zone where both the schistosoma infect rate of residents and livestock were less than 5 %, were no acute infections, and spread-control area refers to the zone where both the schistosoma infect rate were less than 1 %, were no acute infections and no infected *Oncomelania hupensis* snails for two consecutive years [45]. Consequently, a total of four counties was sampled. Second, two townships were randomly selected from each of the four selected counties, yielding a total of eight townships. Third, one middle school in each selected township were randomly sampled. Fourth, three classes per grade in each selected middle school were randomly sampled. Seventy-two classes in total were included. All the students in the selected classes were recruited in this study.

Among the total 2796 participants, 274 (9.80 %) declined to participate. Among the remaining 2522 participants, 284 (10.20 %) were excluded due to missing data on key variables (age, gender, perceptions of harm from schistosomiasis), yielding a final sample of 2238 (80.0 %). There was no significant difference in the key study variables among those who were excluded and those included in the study.

### Data collection and procedures

Access to each sampled school was obtained from the school administration with the assistance of the local Anti-Schistosomiasis Stations in the sampled county. Data were collected using the Adolescents Health and Behavior Survey (AHBS). It took approximately 20 min for most students to complete the questionnaire.

The survey was administrated by eight trained graduate students from School of Public Health, Wuhan University. With the help of the local county Anti-Schistosomiasis Stations and the sampling school, students were gathered in a designate classroom. The survey was anonymous and confidential. The students were asked to complete the questionnaires independently. They were also allowed to skip questions they did not want to answer.

### Scale development

Several steps were followed: (1) the team members collaborated to review all the related literature and generate the constructs of the PMT matching with the schistosomiasis prevention and control. (2) With the concept mapping technique [46] and the literature review [25–27, 38, 41, 44], the designer of this program initially proposed a 26-item instrument with seven sub-scales, including severity (a typical item is “My whole family will suffer if I am infected with schistosomiasis”), vulnerability (a typical item is “I will get infected if I contact snail-conditioned water while assisting my parents to work”), intrinsic reward (a typical item is “It is very enjoyable for me to play in water outside”), extrinsic reward (a typical item is “Washing my hands and feet or clothes in river and ponds will save tap water, save money for my family”), response efficacy (a typical item is “I will never be infected if I do not play in snail-conditioned water”), self-efficacy (a typical item is “I can control myself not to go and play in snail-conditioned water”), and response costs (a typical item is “My friends may tease me if I refuse to go out with them to play in snail-conditioned water”). These items were assessed using a 5-point Likert scale with1 = *definitely agree* to 5 = *definitely disagree*. (3) The draft was circulated twice among the team members, schistosomiasis experts, and graduate students. The items with low correlation with total scale were removed from the scale. A pilot version of the instrument was produced. The scale consisted of 20 items while seven sub-constructs (severity, vulnerability, intrinsic reward, extrinsic reward, response efficacy, self-efficacy, response costs), and each contains 2–3 items. (4) The exploratory factor analysis was conducted. Eigenvalues more than 1 were utilized for choosing the number of factors, and factor loadings of 0.4 or above were considered acceptable [47]. Eventually, two items below 0.4 were eliminated and the final instrument contained 18 items.

### Variables assessing behavior intention

The behavior intention was assessed based on the participants’ response to the question “I am sure I will not play or work in the snail-conditioned water” and “I am sure I will take protective measure when contacting water which is likely to be contaminated by snails” in both 3-month and 12-month period. A five-point Likert scale with “1 = Strongly disagree” and “5 = Strongly agree” was used to assess the behavior intention.

### Demographic variables

Demographic variables included: age (in years), gender (male/female), grade (grade 7–9), if single child in family (yes/no) and parents’ education (primary or less, middle school, and high school or more).

### Statistical analysis

The exploratory factor analysis was conducted by using the principal component analysis with varimax rotation. Item response was assessed using mean and standard deviation. Internal consistency was assessed using the item-to-total correlation. Reliability was assessed using the Cronbach’s alpha for the whole scale, as well as the sub-constructs. To assess the construct validity, we used the Confirmatory Factor Analysis to analyze the data with a criteria of good model fit (GFI > 0.9, CFI > 0.9, RMSEA < 0.05, Chisq/df < 2). Pearson correlation was used to examine the association between PMT sub-constructs and short-term and long-term intention, and the significance of correlation coefficients was the criteria for predictive validity. All analysis were conducted with the software SAS version 9.3 (SAS Institute Inc. Cary, NC). The SPSS version 19.0 (SPSS, Inc., Chicago, IL) software package was used for the Bartlett’s Test of Sphericity and KMO test. TypeIerror was set at 0.05 (two-side).

## Results

### Sample characteristics

Among the 2238 students, 1138 (51 %) were male and the mean age was 13.13 years (SD = 1.10). The proportion of students in the three grades was approximately the same with 31, 37 and 32 % respectively. More than 60 % of the participants reported that their parents’ educational level was middle school (Table 1).

**Table 1:** Characteristics of The Study Sample

Variables	Male	Female	Total
Total, *n* (%)	1138 (50.85)	1100 (49.15)	2238 (100.00)
Chronological Age			
Mean (SD)	13.22 (1.12)	13.03 (1.07)	13.13 (1.10)
School grade, *n* (%)			
Grade 7	359 (31.55)	339 (30.82)	698 (31.19)
Grade 8	415 (36.46)	410 (37.27)	825 (36.86)
Grade 9	364 (31.99)	351 (31.91)	715 (31.95)
If single child, *n* (%)			
Yes	702 (61.69)	500 (45.45)	1202 (53.71)
No	436 (38.31)	600 (54.55)	1036 (46.29)
Father’s education			
Primary or less	160 (14.06)	160 (14.55)	320 (14.30)
Middle school	742 (65.20)	719 (65.36)	1461 (65.28)
High school or more	236 (20.74)	221 (20.09)	457 (20.42)
Mother’s education			
Primary or less	223 (19.60)	241 (21.91)	464 (20.73)
Middle school	705 (61.95)	672 (61.09)	1377 (61.53)
High school or more	210 (18.45)	187 (17.00)	397 (17.74)

### Item response

The results of principle component analysis after deleting two ineligible items in Table 2 showed that the rotated factor loads of items varied from 0.65 to 0.95, and the 7 factors explained 72.8 % of the observed variance. The mean score of each item was ranging from 1.7 to 4.4. The correlation with total varied between 0.11 and 0.57 (Table 3).

**Table 2:** Results of the Rotated Factor Loading Analysis (Varimax Rotated)

Item	Factor1	Factor2	Factor3	Factor4	Factor5	Factor6	Factor7
Q1							0.83
Q2							0.82
Q3		0.85					
Q4		0.87					
Q5		0.81					
Q6				0.86			
Q7				0.82			
Q8				0.56			
Q9	0.70						
Q10	0.84						
Q11	0.83						
Q12					0.95		
Q13					0.95		
Q14			0.65				
Q15			0.87				
Q16			0.83				
Q17						0.84	
Q18						0.85	
Eigen values	2.26	2.22	1.96	1.93	1.83	1.53	1.39
Explained variance (%)	12.54	12.36	10.87	10.69	10.15	8.48	7.74
Cumulative variance (%)	12.54	24.90	35.77	46.46	56.61	65.09	72.83

**Table 3:** Items of 18-Item Instrument Based on PMT for predicting Intention to Engage in Protective Behaviors against Schistosomiasis among middle school students in rural China

Item by constructs	Mean (SD)	r with total
Q1. My whole family will suffer if I am infected with schistosomiasis	2.87 (1.22)	0.29
Q2. I will become hopeless for the rest of my life if I am infected with schistosomiasis.	2.43 (1.16)	0.31
Q3. I will get infected if I contact snail-conditioned water while assisting my parents to work.	4.32 (0.84)	0.15
Q4. I will get infected if I play with or swim in snail-conditioned water.	4.39 (0.81)	0.18
Q5. If I wade through water or walk in wetland in snail-conditioned areas without protection, I will get infected.	4.36 (0.83)	0.15
Q6. It is very enjoyable for me to play in water outside.	2.05 (1.26)	0.47
Q7. It is convenient to wash my hands and feet in rivers and ponds.	1.99 (1.25)	0.51
Q8. It will be more convenient to assist parents’ work or play in snail-conditioned water without protective measures.	2.02 (1.31)	0.49
Q9. Playing in outdoor water with classmates is good for making friends and for strengthening friendship.	1.90 (1.10)	0.53
Q10. Washing my hands and feet or clothes in river and ponds will save tap water, save money for my family.	1.90 (1.14)	0.57
Q11. It will save money if assisting parents’ work or playing in water without protection (water shoes, protective gels such as plant cercaricide Fangyouling).	1.72 (1.01)	0.56
Q12. I will never be infected if I do not play in snail-conditioned water.	2.76 (1.14)	0.44
Q13. I will never get infected if I do not assist parents’ work in snail-conditioned water.	2.80 (1.12)	0.44
Q14. I can control myself not to go and play in snail-conditioned water.	4.20 (1.02)	0.17
Q15. I can definitely say “no” even if my friends invite me to play in snail-conditioned water.	4.47 (0.92)	0.11
Q16. I can definitely say “no” even if my parents or teachers ask me to do things that may involve snail-conditioned water.	4.38 (0.98)	0.11
Q17. My friends may tease me if I refuse to go out with them to play in snail-conditioned water	2.15 (1.28)	0.43
Q18. My parents will scold me if I worry about schistosomiasis and refuse to do things in snail-conditioned water.	1.86 (1.15)	0.42

### Reliability

Results in Table 4 indicated that the Cronbach’s Alpha was 0.76 for the PMT instrument. And the alpha for 7 sub-constructs varied between 0.56 and 0.90. The reliability was also assessed by gender and school grade.

**Table 4:** Cronbach’s α of the PMT instrument, overall and by subsample

Variables	PMT	S	V	IR	ER	RE	SE	RC
Total sample	0.76	0.56	0.82	0.75	0.80	0.90	0.72	0.70
Gender								
Male	0.75	0.55	0.81	0.77	0.80	0.88	0.69	0.70
Female	0.75	0.57	0.84	0.71	0.78	0.93	0.74	0.67
School grade								
Grade 7	0.77	0.54	0.82	0.76	0.80	0.89	0.72	0.70
Grade 8	0.75	0.52	0.79	0.75	0.78	0.89	0.73	0.68
Grade 9	0.74	0.61	0.84	0.73	0.80	0.92	0.72	0.71

Note: *PMT*: the PMT instrument, *S*: severity, *V*: vulnerability, *IR*: intrinsic reward, *ER*: extrinsic reward, *RE*: response efficacy, *SE*: self-efficacy, *RC*: response cost

### Construct validity

**Fig. 2:**
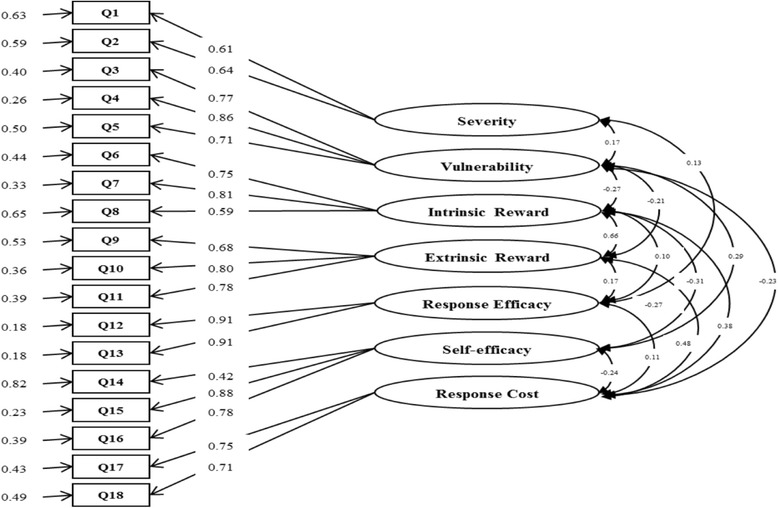
One-level Seven-Factor Model for the Instrument Based on PMT

Results in Fig. 2 illustrated one-level and 7-subconstruct models of the 18-item instrument, and the model fit the data well (GFI > 0.9, CFI > 0.9, RMSEA = 0.03, chi-square/df = 3.90). Each item was highly related to its sub-construct and the model coefficients varied from 0.42 to 0.91.

### Predictive validity

Table 5 summarizes the correlation between behavioral intention and PMT instrument. The PMT scores of all sub-constructs are significantly associated with the short and long-term protective behavioral intention against schistosomiasis. In addition, Severity (0.05, 0.05), Vulnerability (0.20, 0.21), Response Efficacy (0.06, 0.07) and Self-Efficacy (0.40, 0.42) were positively related to intention, whereas Intrinsic Reward (−0.12, −0.15), Extrinsic Reward (−0.12, −0.14) and Response Cost (−0.12, −0.14) were negatively associated with protective behavior intention. Among these sub-constructs, Self-Efficacy has the high coefficient, followed by Vulnerability.

**Table 5:** Correlations of the Instrument Based on PMT and Protective Behavioral Intention

PMT Subconstruct	Short-term Behavior Intention	Long-term Behavior Intention
Severity (S)	0.05*	0.05*
Vulnerability (V)	0.20**	0.21**
Intrinsic Reward (IR)	−0.12**	−0.15**
Extrinsic Reward (ER)	−0.12**	−0.14**
Response Efficacy (RE)	0.06**	0.07**
Self-Efficacy (SE)	0.40**	0.42**
Response Cost (RC)	−0.12**	−0.14**

Note: **p < 0.01, *p < 0.05

## Discussion

In this study, we reported a tool developed based on Protective Motivation Theory (PMT) to predict protective behavior intention against schistosomiasis. Concept mapping was used to draft the scale. Cronbach alpha was used to measure the reliability, Confirmatory Factor Analysis was used to assess the construct validity, and correlation analysis was used to assess predictive validity. Findings in this study indicated that the instrument was reliable and valid, and could be used for future study.

The Cronbach’s Alpha for overall PMT instrument is good (0.76). Moreover, for 6 of the 7 sub-constructs, Cronbach’s Alphas in total sample are greater than 0.7, and in each subsample, most Cronbach’s Alphas are still greater 0.7 and the rest are very close to 0.7; thus the performances are good for the sub-constructs Vulnerability, Intrinsic reward, Extrinsic reward, Response efficacy, Self-efficacy and Response cost. The only questionable sub-construct is Severity, the results for this sub-construct is relatively poor, but considering the small number of items in this sub-construct (*n* = 2), it is still close to the acceptable level (alpha = 0.56). Further studies were needed in the future.

The results from Confirmatory Factor Analysis (CFA) demonstrated that the 18-item instrument was validated for applying Protection Motivation Theory (PMT) in research on behavioral intention relating to schistosomiasis. The indexes from the CFA indicated the 7-factor model fit the data well, and the outcome is similar to the applications of PMT in other health issues [26, 36, 38]. The sub-constructs Severity, Vulnerability, Response Efficacy and Self-Efficacy are positively correlated with the protective behavioral intention against schistosomiasis while Intrinsic Reward, Extrinsic Reward and Response cost are negatively correlated, and these outcomes were consist with the original hypothesis of Protection Motivation Theory [27, 36, 48].

Behavioral study showed that the intention of human being was generally regarded as the most vital determinant of the behavior [24]. Therefore, this instrument may be effective for both etiological and applied studies of schistosomasis control and prevention. As a social cognitive conceptual theory, the PMT emphasized assessing the risks of diseases, benefits from protection and motivations to protective behaviors [48, 49]. All the seven sub-constructs were potential independent, mediating and/ or outcome variables when developing and evaluating interventions [26, 44]. Hence in etiological studies, the instrument can be used to measure these potential variables and analyze the mechanism how the interventions work. On the other hand, from the applied perspective, the predictive validity showed that the sub-constructs were significantly correlated with the protective behavioral intention. This finding suggests that the following interventions should include focusing on Chinese adolescents’ cognition (knowledge) about the severity and vulnerability of schistosomiasis, improving response efficacy and self-efficacy of protective behavior against schistosomiasis, as well as decreasing the intrinsic and extrinsic reward, and response cost at the same time [35, 44]. More specific interventions should be developed to promote protective behaviors. In addition to the current health education which mainly focuses on the knowledge delivery, we could develop particular programs to improve individuals’ protective motivation. First, the contents of health education could be upgraded. For example, more information about the validity of the protection materials should be added. Second, the self-efficacy of the adolescents should be improved. For example, we could train the students to just say “no” to the schistosomiasis related risk behaviors, such as playing in the tail contaminated water, working in the farm without protection. An integrated intervention program that incorporate the above information and skills will be helpful to reduce the incidence of schistosomiasis and improve the quality of life of the people living in the tail contaminated area.

There are also limitations in this study. First, the data for psychometric assessment were collected from rural areas in Hubei Province, hence the heterogeneity among areas that might limit the general application in China. Second, the protective behavioral intention, on behalf of protective behaviors against schistosomasis actually, was self-reported. It is hard to exclude information bias since the adolescents might report the intention dishonestly or behave unintentionally. More objective measurements of protective behaviors such as videotaping or GIS positioning technology could be applied in the further study [21]. Third, the result of reliability test for sub-construct Severity was not as good as others, so the items in this sub-construct could be improved. Fourth, test-retest was not conducted, thus further study could include a validate study for stability. Last but not the least, the PMT theory may not explain the non-rational component of behaviors, limiting the wide application of PMT theory in predicting risk behaviors.

In spite of the limitations above, this study was the first to develop an instrument based on PMT in predicting Chinese adolescents’ Intention to engage in protective behaviors against schistosomasis. It is fundamental to apply PMT on schistosomasis control and prevention. Besides revising the instrument, further studies can focus on exploring the mechanism of PMT on protective behavior against schistosomasis, such as how the sub-constructs influence the protective behavior together with other social and individual factors, and how the influence varies in time, or the intervention trial based on the PMT.

## Conclusion

In conclusion, the psychometrical measurement suggests that the instrument was a valid and reliable tool for predicting Chinese adolescents’ intention to engage in protective behaviors against schistosomiasis, indicating Protection Motivation Theory was promising for schistosomiasis control and prevention.

## Abbreviations

AHBS: the Adolescents Health and Behavior Survey
CFA: Confirmatory Factor Analysis
CFI: Comparative Fit Index
GFI: Goodness of Fit Index
GIS: Geographic Information System
HIV/AIDS: Human Immunodeficiency Virus/Acquired Immunodeficiency Syndrome
KAP: Knowledge-Attitude-Practice
KMO: Kaiser-Meyer-Olkin Test
PMT: Protection Motivation Theory
RMSEA: Root Mean Square Error of Approximation
